# *M*. *tuberculosis* infection and antigen specific cytokine response in healthcare workers frequently exposed to tuberculosis

**DOI:** 10.1038/s41598-019-44294-0

**Published:** 2019-06-03

**Authors:** Paulin N. Essone, Marielle Leboueny, Anicet Christel Maloupazoa Siawaya, Amel Kévin Alame-Emane, Oriane Cordelia Aboumegone Biyogo, Patrice Hemery Dapnet Tadatsin, Amandine Mveang Nzoghe, Dimitri Ulrich Essamazokou, Ofilia Mvoundza Ndjindji, Guy-Stéphane Padzys, Selidji Todagbe Agnandji, Howard Takiff, Brigitte Gicquel, Joel Fleury Djoba Siawaya

**Affiliations:** 1 Unité de Recherche et de Diagnostics Spécialisés, Laboratoire National de Santé Publique/Centre Hospitalier Universitaire Mère Enfant Fondation Jeanne EBORI, Lambaréné, Gabon; 2 Centre de Recherches Médicales de Lambaréné, BP 242 Lambaréné, Gabon; 3 Unité de Génétique Mycobactérienne, Institut Pasteur, Paris, France; 4 Département de Biologie Cellulaire et Physiologie Faculté des Sciences, Université des Sciences et Techniques de Masuku, Franceville, Gabon; 5 Unité de Pathogenomique Mycobactérienne Intégrée, Institut Pasteur, Paris, France; 6 Department of Tuberculosis Control and Prevention, Shenzhen Nanshan Center for Chronic Disease Control, Shenzhen, China; 7 Institut für Tropenmedizin, Universitätsklinikum Tübingen, Tübingen, Germany

**Keywords:** Tuberculosis, Biomarkers

## Abstract

Tuberculosis (TB) is the leading cause of death due to an infectious agent, but only a small fraction of those infected develop the disease. Cytokines are involved in the mediation and regulation of immunity, and their secretion patterns may reflect the infection status. To increase our understanding of immune response to *M*. *tuberculosis* infection, we conducted a cross-sectional study investigating *M*. *tuberculosis* infection status and comparing the release profiles of cytokines GM-CSF, IFN-γ, IL-1β, IL-10, IL-12 (p70), IL-2, IL-4, IL-5, IL-6, IL-8, TNF-α, in community controls (CCs) and healthy healthcare workers (HCWs) highly exposed to TB. Among HCWs and CCs, the probability of latent *M*. *tuberculosis* (LTB^+^) infection was respectively 5.4 (p = 0.002) and 3.4 (p = 0.006) times higher in men than women. The odds ratio of LTB infection was 4 times higher among HCWs in direct contact with active TB patients than other HCW (p = 0.01). Whole blood supernatant cytokine responses to *M*. *tuberculosis* antigens showed differential pro-inflammatory responses between HCWs and CCs. CCs^LTB−^ had higher IL-1β responses than HCWs^LTB−^ (p = 0.002). HCWs^LTB+^ had significantly higher IL-8 responses to *M*. *tuberculosis* antigens than HCWs^LTB−^ (p = 0.003) and CCs^LTB−^ (p = 0.015). HCWs^LTB+/−^ showed weak but positive TNF-α responses to *M*. *tuberculosis* antigen stimulation compared to CCs^LTB+/−^ (p ≤ 0.015). Looking at T-helper (1 and 2) responses, HCWs^LTB+^ and CCs^LTB+^ had significantly higher IFN-γ and IL-2 responses compared to HCWs^LTB−^ and CCs^LTB−^ (p < [0.0001–0.003]). Also, TB antigen induced IL-5 secretion was significantly higher in HCWs^LTB+^ and CCs^LTB+^ than in non-infected CCs^LTB−^ (p < [0.005–0.04]). *M*. *tuberculosis* antigen specific responses in HCWs^LTB+^ varied based on active TB exposure gradient. HCWs^LTB+^ who were highly exposed to active TB (≥3 hours per day) had significantly higher IFN-γ and IL-8 responses (p ≤ 0.02) than HCWs ^LTB+^ not in direct contact with active TB patients. HCWs^LTB+^ working with active TB patients for 5 to 31 years had a significantly enhanced secretion of proinflammatory cytokines (GM-CSF, IFN-γ, IL-1β, IL-2, IL-6, IL-8, IL-12p70, TNF-α) compared to HCWs^LTB−^ (p < [0.0001–0.01]). Secretion of anti-inflammatory/Th2 cytokines IL-5 and IL-10 was also higher in HCWs^LTB+^ than HCWs^LTB−^. In conclusion, LTBI individuals controlling the *M*. *tuberculosis* infection have an enhanced TB specific Th1-cytokines/proinflammatory response combined with selected Th2 type/anti-inflammatory cytokines induction.

## Introduction

One third of the global population is infected by *Mycobacterium tuberculosis* but only 5 to 10% will ever develop the disease^1^. TB infection is particularly prevalent in developing countries characterized by limited health budgets. A preventive prophylaxis policy has been introduced to prevent the *M*. *tuberculosis* infected population, as determined by the tuberculin skin test (TST) or an interferon gamma release assay (IGRA), from progressing to active TB. However, this policy cannot be applied in high incidence developing countries due to the projected high cost, associated toxicities, the possibility of anti-TB drug resistance and the high risk of re-infection after prophylactic therapy. With more than 10 million new cases of TB per year worldwide, TB continues to be a major global health problem^2^. Despite availability of a partially effective vaccine and a reasonably effective but long drug therapy, this disease causes more than one (1) million deaths every year^2^. Accurate and accessible diagnostic tools, the introduction of shorter TB therapy and a more effective vaccine are all needed to make substantial progress towards reducing the global burden of TB.

Although protective immunity against *M*. *tuberculosis* is not completely understood, we know that the host control of *M*. *tuberculosis* infection depends on both innate and adaptive immune mechanisms^3,4^. After *M*. *tuberculosis* inhalation, the innate immune system in some people can clear the infection before the initiation of the adaptive immune response that is characteristic of *M*. *tuberculosis* infection^4,5^, but little is known about the specific mechanisms that enable this early clearance of *M*. *tuberculosis*. It has been suggested that a robust innate immune responses or the rapid activation of macrophages is required to clear an incipient *M*. *tuberculosis* infection^6^, including the recognition of *M*. *tuberculosis* surface antigens by airway epithelial cells (AECs), and the production of interferon (IFN)‐γ, tumour necrosis factor (TNF)‐α, granzyme, β-defensin 2, cathelicidin, and hepcidin. Granulocytes are also thought to have a role in protecting against *M*. *tuberculosis* infection^7^.

The dynamic process leading to latent TB (LTB) infection is better described than the mechanisms that mediate resistance to *M*. *tuberculosis* infection. After *M*. *tuberculosis* entry, the alveolar macrophages produce inflammatory cytokines and chemokines that signal infection. The monocytes, neutrophils, and lymphocytes migrate to the infection site, and this collection of inflammatory cells forms an immune and physical barrier (termed granuloma) to contain the infection and prevent *M*. *tuberculosis* dissemination^8^. Th1-type and pro-inflammatory cytokines (eg: IFNγ, IP-10, RANTES, TNF‐α, and IL-12) are involved in forming and maintaining of the integrity of the granuloma^9–11^. However, knowing the key players in TB is not sufficient, as Walzl and colleagues clearly highlighted: *“The immune responses that are crucial for protection against clinically active M*. *tuberculosis infection may not necessarily translate into correlates of protection or risk in humans*^12^”. The present study aimed to assess the risk of LTB infection in Health Care Workers (HCWs) and to profile the immune system of individuals who are highly exposed to *M*. *tuberculosis* but resist or control the infection. We hypothesized that an analysis of the immune profile of highly exposed non-infected individuals might help identify immune markers of resistance to *M*. *tuberculosis* infection^12^. Similarly, the immune profile of latently infected healthy individuals could yield information on how their *M*. *tuberculosis* infection is controlled and maintained in a latent state. Thus, in a targeted hypothesis-driven approach, we investigated the immune response of non-infected and latently infected HCWs  who have been highly exposed to *M*. *tuberculosis*.

## Methods

### Study design and participants

Using a cross sectional approach, we recruited 76 healthcare workers (HCWs) and 93 healthy community controls (CCs). Our recruitment possibilities were limited by the reduced number of human resources assigned to institutions specialized in the care of tuberculosis. All participants were HIV-negative. Of the 76 HCWs, 55 workers came from the Nkembo TB Specialized Hospital in Libreville (Gabon), which sees approximately 3900 new notified TB cases per year, but has closed or poorly ventilated consultation rooms, no ultraviolet or germicidal irradiation, and poor adherence of workers to health protection measures such as respiratory protective equipment. Twenty HCWs were from the National Laboratory of Public Health in Libreville, which has a biosafety level 2 TB bacilloscopy laboratory and low direct exposure to TB patients, and one HCW was from the emergency medical service. CCs were recruited from the community. From all participants we collected anthropometric data (age, sex, height and weight) and the following information: the place of work, work description, number of years in service, number of hours per day spent in contact with TB patients, TB-history (previous exposure to active TB household case), BCG vaccination, alcohol consumption, cigarette consumption and chronic diseases. Participants with any suggestion of active TB, including fever, weight loss, prolonged fatigue or any other clinical sign of disease, were excluded from the study. Also excluded were participants with a previous history of active TB. A signed informed consent was obtained from all participants. The height and weight of participants were measured using a measuring rod and a body weight scale. The body mass index (BMI), was calculated as the body weight divided by the square of the body height, and expressed in units of kg/m^2^. The research was done in accordance with Gabonese ethical guidelines and regulations, and approval was obtained from the Gabonese National Laboratory of Public Health ethics committee. The Institute Pasteur Center for Translational Research (CRT) open desk also approved the study.

### Latent TB infection diagnosis

All participants were screened with the QuantiFERON -TB (QFT) Gold in-tube test, (QIAGEN - France), following the manufacturer’s guidelines. Participants with positive tests were considered to have a latent TB infection (LTB).

### Participants’ classification base on active TB exposition

An exposure gradient with three levels was defined for individual HCW according to the number of hours spent per day with TB patients. High exposure: participants working 3 successive hours or more per day with active TB-patients or within their direct environment. Moderate exposure: participants working 1–2 hours per day with active TB-patients or within their direct environment. Low exposure: not working in an environment with active TB patients. Highly exposed subjects with a negative IGRA result were assumed to have cleared a possible *M*. *tuberculosis* infection^4^ or to be resistant to infection with *M*. *tuberculosis*. Due to the small numbers of moderately exposed HCWs, study participants were grouped into six (6) categories: (1) highly exposed, latently infected HCWs; (2) highly exposed, non-infected HCWs; (3) low exposure, latently infected HCWs; (4) low exposure non-infected HCWs; (5) latently infected CCs; (6) non-infected CCs.

### Cytokines secretion in response to TB-antigens stimulation

#### Sample handling

Whole blood was collected from all study participants into QFT test tubes including the Nil, Mitogen and *M*. *tuberculosis*-antigen (ESAT-6/CFP-10/TB7.7) tubes. The tubes were then incubated overnight in a humidified incubator at 37 °C, with 5% CO_2_. The following day, supernatants were harvested, aliquoted and stored at −40 °C until cytokine measurement.

#### Luminex analysis

The determination of cytokine concentrations in whole blood supernatant from participants was done with the Bio-Plex 200 bead array system (Bio-Rad Laboratories, USA). Assays were carried out in 96-well filter plates using the Procartaplex11-plex from Life Technologies -Thermo Fisher Scientific (USA). The cytokines assayed, according to the manufacturer’s instructions, were: GM-CSF, IFN-γ, IL-1β, IL-10, IL-12 (p70), IL-2, IL-4, IL-5, IL-6, IL-8, and TNF-α.

#### Determination of cytokines responses

*M*. *tuberculosis* antigen induced cytokine concentrations were determined by subtracting baseline cytokines concentrations (nil tube) from the concentration of cytokines measured in the TB-antigen tube.

### Statistical analysis

The statistical analysis was performed using GraphPad Prism software version 6. A chi-square test was used to compare the frequencies of test results among different groups of participants. Because it is known that BMI, social conditions, behaviors and chronic illness influence the risk of TB infection and constitute a potential sources of bias; we assessed the association between TB infection and those variables using the contingency table from which odds ratios (OR) and 95% confidence intervals (CI) were derived.

The Mann-Whitney U-test was performed to compare two groups. When more than two groups were compared, we used the ANOVA one-way non-parametric multiple comparisons test (Kruskal-Wallis test) coupled to the Dunn’s multiple comparisons test. The threshold of significance was a p-value below 0.05.

## Results

The characteristics of the studied populations are described in Table 1. Both HCWs and CCs were predominantly female (77% and 67% respectively). Anthropometric data (age and BMI) of HCWs and CCs were comparable (see Table 1). However, gender analysis showed that the BMI of male HCWs was significantly lower than that of female HCWs (Supplementary Figure), while male and female CCs had comparable BMI’s. The percentage of participants having received BCG vaccination was high in both HCWs (83%) and CCs (90%). Half of HCWs and 41% of CCs declared alcohol consumption, but the percentage of cigarette smokers was low in both HCWs and CCs (8% and 3% respectively). The percentage of study participants with TB household contacts was also low, 5% of HCWs and 2% of CCs.

**Table 1:** Characteristics of the studied populations, rate and odds of latent TB infection.

Variables	Healthcare workers			Community Controls		
	*N (%)*	*Positive Quantiferon Test (%)*	*Odds ratios (OR) /Relative risk (RR) [Chi-square test p-value]* at 95%CI	*N (%)*	*Positive Quantiferon Test (%)*	*Odds ratios (OR) /Relative risk (RR) [Chi-square test p-value]* at 95%CI
All	75 (100)	24 (32)		93 (100)	29 (31)	
**Gender**						
Female	58 (77)	13 (22)	0.16/0.3 [0,001] as compared with male	62 (67)	15 (24)	0.29/0.43 [0,006] as compared with female
Male	17 (23)	11 (65)	6/3 [0,001] as compared with female	31 (33)	14 (45)	3.4/2.3 [0,006] as compared with female
**Age**						
<35 years	19 (25.3)	6 (30)	1/1 [0.95] as compared with the rest of participant	49 (53)	16 (33)	1.16/1.1 [0.75] as compared with the rest of participant
35–45 years	34 (42.3)	32 (11)	1/1 [0.95] as compared with the rest of participant	18 (19)	7 (39)	1.5/1.3 [0.43] as compared with the rest of participant
≥45 years	22 (29.3)	32 (7)	1/1 [1] as compared with the rest of participant	26 (28)	6 (23)	0.57/0.67 [0.29] as compared with the rest of participant
**Body mass index (BMI)**						
Median BMI (Min - Max)	27 (16–44)	26 (18–40)	—	25 (16–42)	25 (19–44)	—
Percentile [25%- 75%]	[24–32]	[22–34]	—	[22–29]	[22–28]	—
Female	30 (16–42)	31 (18–40)		25.4 (19–44)	24 (21–38.5)	
Male	24 (19–43)	23 (19–29)		25.5 (17–42)	25 (17–31)	
**Job designation/Occupation**						
Administration	10 (13)	1 (10)	0.2/0.3 [0.16]	—	—	—
Laboratory technicians	12 (16)	2 (25)	0.7/0.75 [0.74] as compared with other health workers	—	—	—
Medical doctors and nurse *	23 (31)	8 (35)	1/1 [1] as compared with other health workers	—	—	—
Medical secretaries and Receptionists (patients’ desk) *	10 (13)	6 (60)	4/2 [0.06] as compared with other health workers	—	—	—
Radiology technicians*(!)	3 (4)	(3) 100	17/3 [0.03] as compared with other health workers	—	—	—
Social workers	6 (8)	0 (0)	0.3/0.4 [0.4]		—	—
Support staff/cleaners	11 (15)	3 (27)	0.8/0.83 [1]			
Workers in direct day-to-day contact with patients (*) [Highly exposed]	36 (48)	17 (47)	4/3 [0.01] as compared with other health workers			
				—	—	—
Formal workers (lawyers, law enforcement officers, accountant, teachers, biologists, etc.)	—	—	—	23 (25)	9 (39)	1.6/1.4 [0.3] as compared with other groups of workers and non-workers
Informal sector (traders, drivers, etc.)	—	—	—	12 (13)	6 (50)	2.5/1.8 [0.19] as compared with other groups of workers and non-workers
Students	—	—	—	32 (34)	10 (31)	1/1 [1] as compared with other groups of workers and non-workers
Without a job	—	—	—	26 (28)	4 (15)	0.3/ 0.4 [0.04] as compared with workers and students
**HIV**						
HIV-infected	1 (1)	0 (0)	—	2 (2)	1 (50)	—
Non-HIV-infected	74 (99)	24 (32)	—	91 (98)	28 (32)	—
**History of BCG**						
BCG	62 (83)	19 (31)	0.3/ 0.5 [0.1] as compared with NO BCG	82 (90)	25 (35.5)	0.4/ 0.6 [0.3] as compared with NO BCG
NO BCG	9 (12)	5 (55.5)	3/2 [0.1] as compared with BCG	9 (10)	3 (33)	2.3/1.6 [0.3] as compared with BCG
Information not provided	4 (5)	0 (0)	—	2 (2)	1(50)	—
**Household TB-contact**						
NO	45 (60)	15 (33)	0.17/0.44 [0.09] as compared with TB-contact	76 (82)	23 (30)	0.4/ 0.0 [0.3] as compared with TB-contact
YES (!)	4 (5)	3 (75)	6/2.2 [0.09] as compared with non-contact	2 (2)	0 (0)	—
Information not provided	26 (35)	6 (23)	—	15 (16)	6 (40)	—
**Years working in TB patients care**						
0–4	24 (32)	7 (24)	0.7/0.8 [0.8]	—	—	—
5–9	12 (16)	2 (17)	0.3/0.4 [0.1]	—	—	—
⩾10	18 (24)	9 (50)	3/2 [0.06]	—	—	—
**Alcohol consumption**						
NO	33 (44)	11 (33)	1/1 [1] as compared with drinkers	51 (55)	17 (33)	1.9/1.6 [0.2] as compared with drinkers
YES	39 (52)	13 (33)	1/1 [1] as compared with non-drinkers	38 (41)	8 (21)	0.53/0.63 [0.2] as compared with non-drinkers
Information not provided	3 (4)	0 (0)	—	4 (4)	4 (50)	—
**Smoker**						
NO	67 (89)	22 (33)	1/1 [1] as compared with smokers	86 (92.5)	26 (30)	0.6/0.7 [0.5] as compared with smokers
YES (!)	6 (8)	2 (33)	1/1 [1] as compared with non-smokers	7 (75)	3 (43)	1.7/1.4 [0.5] as compared with non-smokers
Information not provided	2 (3)	0 (0)	—	0 (0)	0 (0)	—
Chronic diseases**						
NO	56 (75)	19 (34)	0.8/0.87 [0.7] as compared with chronically ill	64 (69)	21 (33)	2/1.7 [0.2] as compared with chronically ill
YES	13 (17)	5 (38.5)	1.2/1.1 [0.7] as compared with non-chronically ill	24 (26)	5 (21)	0.5/0.6 [0.2] as compared with non-chronically ill
Information not provided	6 (8)	0 (0)	—	3 (3)	2 (67)	—

**High blood pressure, Rheumatism, and the gastritis were recorded chronic conditions.

(!) very low number of participants.

### The odds of LTB infection is higher in men than women

The frequency of latent TB infection was significantly higher in men than women, both in the HCW and CC. A positive Quantiferon test was found in 65% of male HCWs versus 22% of women HCWs, which translates into a 5.4 times greater odds for males being latently infected with TB compared to female HCWs (p = 0.002). In the CC participants, 45% of men versus 24% of women had positive Quantiferon tests, yielding a 3.4 times greater risk of CC men being latently infected compared with CC women (p = 0.006).

### The rate of latent TB is higher in HCWs workers in direct contact with active TB

Although the overall frequency of latent TB infection was similar in all HCWs (all) and all CCs (respectively 32% and 31%), the percentage of positive Quantiferon tests was greater in the highly exposed HCWs than in CCs (47% and 31% respectively) and was four times higher than in HCWs who were not highly exposed (p = 0.01). Also, the number of years working in a healthcare setting increased the probability of LTB infection. The odds of LTB infection in HCWs in service for more than ten (10) years were 3 times greater than in HCWs with less than ten (10) years of service (Table 1).

### Cytokines levels in HCWs and CCs

We compared cytokine responses in latently infected HCWs (LTB+) with non-infected HCWs (LTB−) and CCs (LTB+ and LTB−). HCWs were divided into groups based on their daily exposure to active TB patients and their latent TB infection status as indicated by their Quantiferon results.

#### Cytokines levels before antigenic stimulation

**Figure 1:**
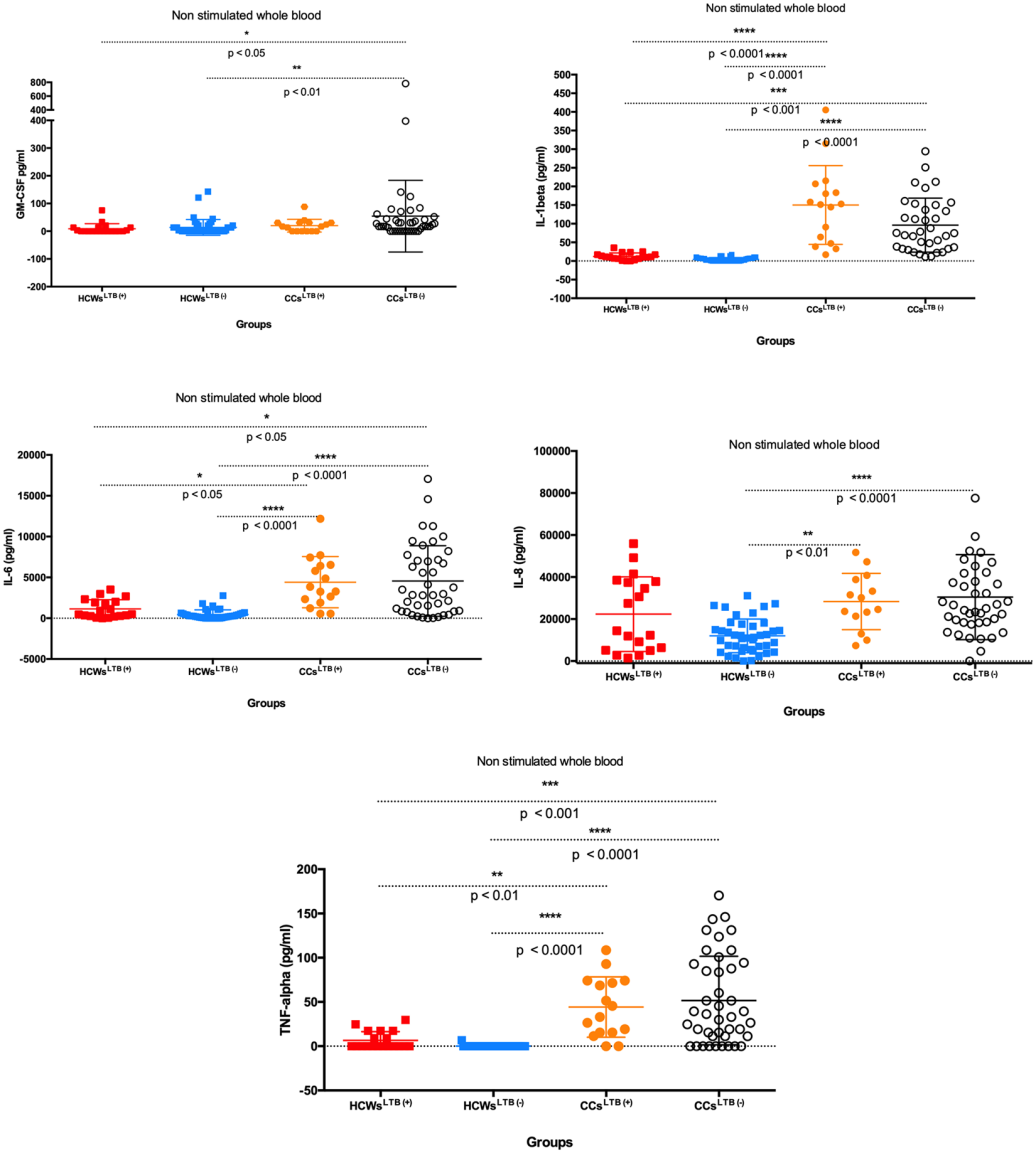
Concentrations of innate immunity and proinflammatory cytokines (GM-CSF, IL-1β, IL-6, IL-8 and TNF-α.) in *M*. *tuberculosis* latently infected (LTB+) and non-infected (LTB−) healthcare workers (HCWs) and community controls (CCs) before *M*. *tuberculosis* antigens stimulation.

The comparison of GM-CSF concentrations across HCWs and CCs groups showed no significant differences between HCWs^LTB+^ and CCs^LTB+^ but CCs^LTB−^ had significantly higher concentrations of GM-CSF than either HCWs^LTB+^ or HCWs^LTB−^ (p < [0.01–0.05]). Both CCs groups (CCs ^LTB+/−^) also had significantly higher concentrations of IL-1β, IL-6 and TNF-α than the two HCWs groups (HCWs^LTB+/−^) (p < [0.0001–0.05]), and IL-8 was significantly higher in CCs^LTB+/−^ compared to HCWs^LTB−^ (p < [0.001–0.01]). No differences in these cytokines were observed between the two HCW groups (Fig. 1).

**Figure 2:**
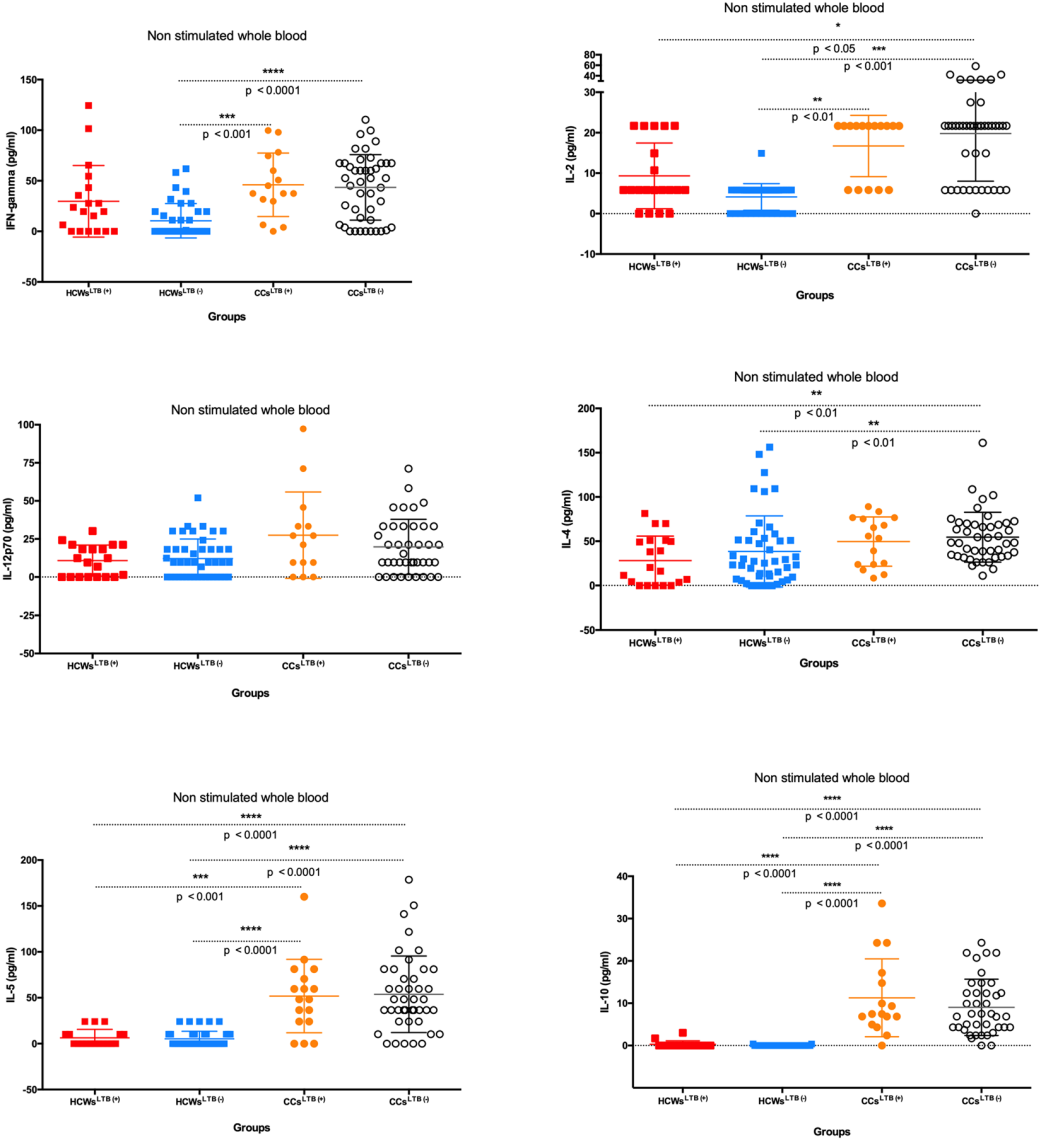
Cell mediated cytokines responses cytokines (IFN-γ, IL-2, IL-12 (p70), IL-4, IL-5 and IL-10) in *M*. *tuberculosis* latently infected (LTB+) and non-infected (LTB−) healthcare workers (HCWs) and community controls (CCs) before *M*. *tuberculosis* antigens stimulation.

There were no significant differences in IL-12p70 between any of the groups. The concentrations of IFN-γ in CCs^LTB+/−^ were significantly higher than in HCWs^LTB−^ (p < 0.0001), although the differences between HCWs^LTB+^ and HCWs^LTB−^ were not significant. IL-2 concentrations were significantly higher in CCs ^LTB+/−^ than HCWs^LTB−^ (p < 0.001), whereas only CCs^LTB−^ had a significantly higher concentration than HCWs^LTB+^ (p < 0.05) (Fig. 2). The concentration of IL-4 in CCs^LTB−^ was significantly higher than in HCWs^LTB+/−^ (p < [0.0001–0.001]) (Fig. 2). No other significant differences in IL-4 were seen between the groups before antigenic stimulation. IL-5 and IL-10 concentrations were significantly higher in both CCs^LTB+/−^ than in the HCWs^LTB+/−^ (p < [0.0001–0.001]) (Fig. 2).

#### Differential cytokines response to M. tuberculosis antigens in HCWs and CCs

**Figure 3:**
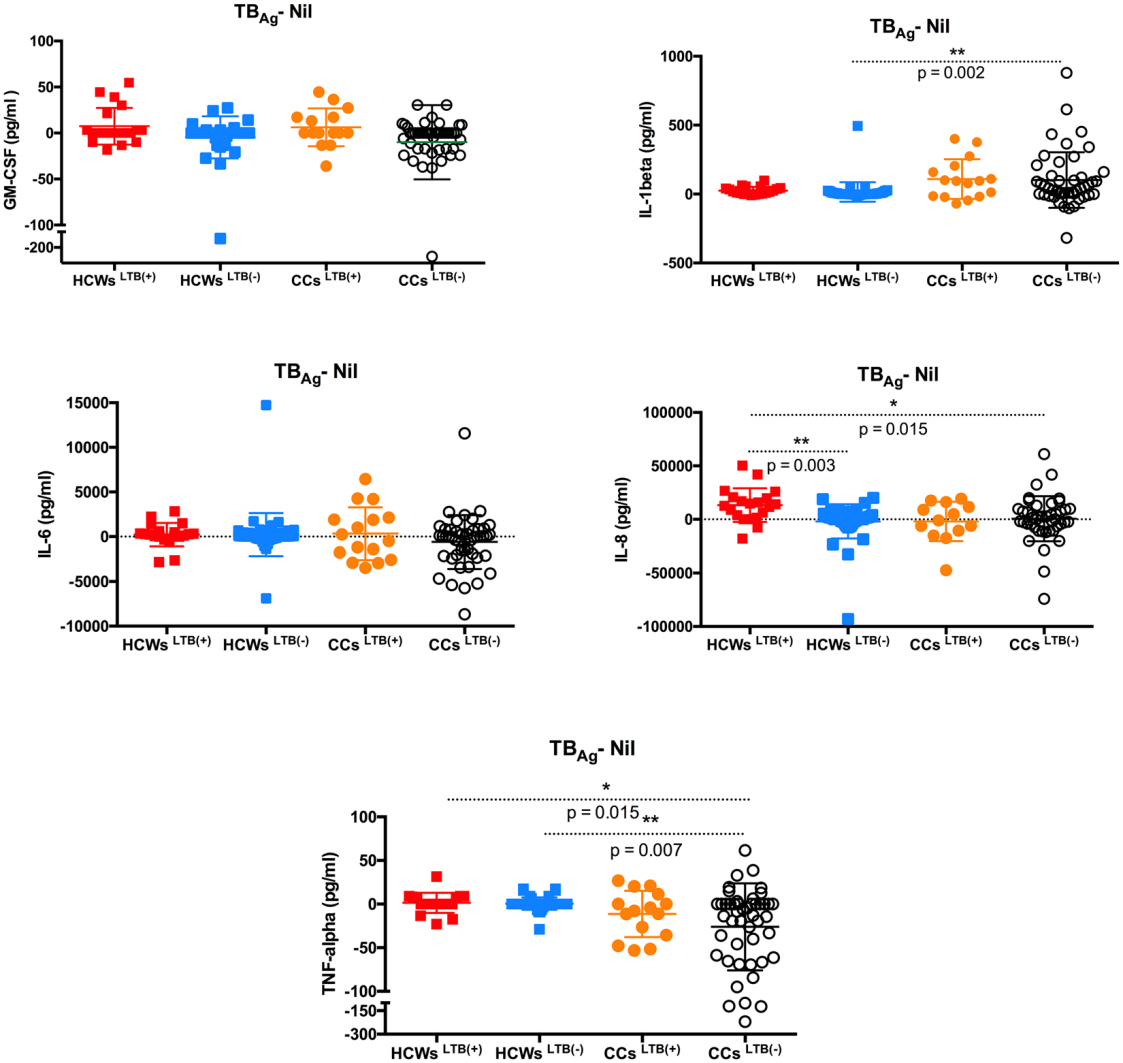
Levels of *M*. *tuberculosis* specific inflammatory markers response (GM-CSF, IL-1β, IL-6, IL-8 and TNF-α) in the supernatants obtained from the QFT® assays of *M*. *tuberculosis* latently infected (LTB+) and non-infected (LTB−) healthcare workers (HCWs) and community controls (CCs).

**Figure 4:**
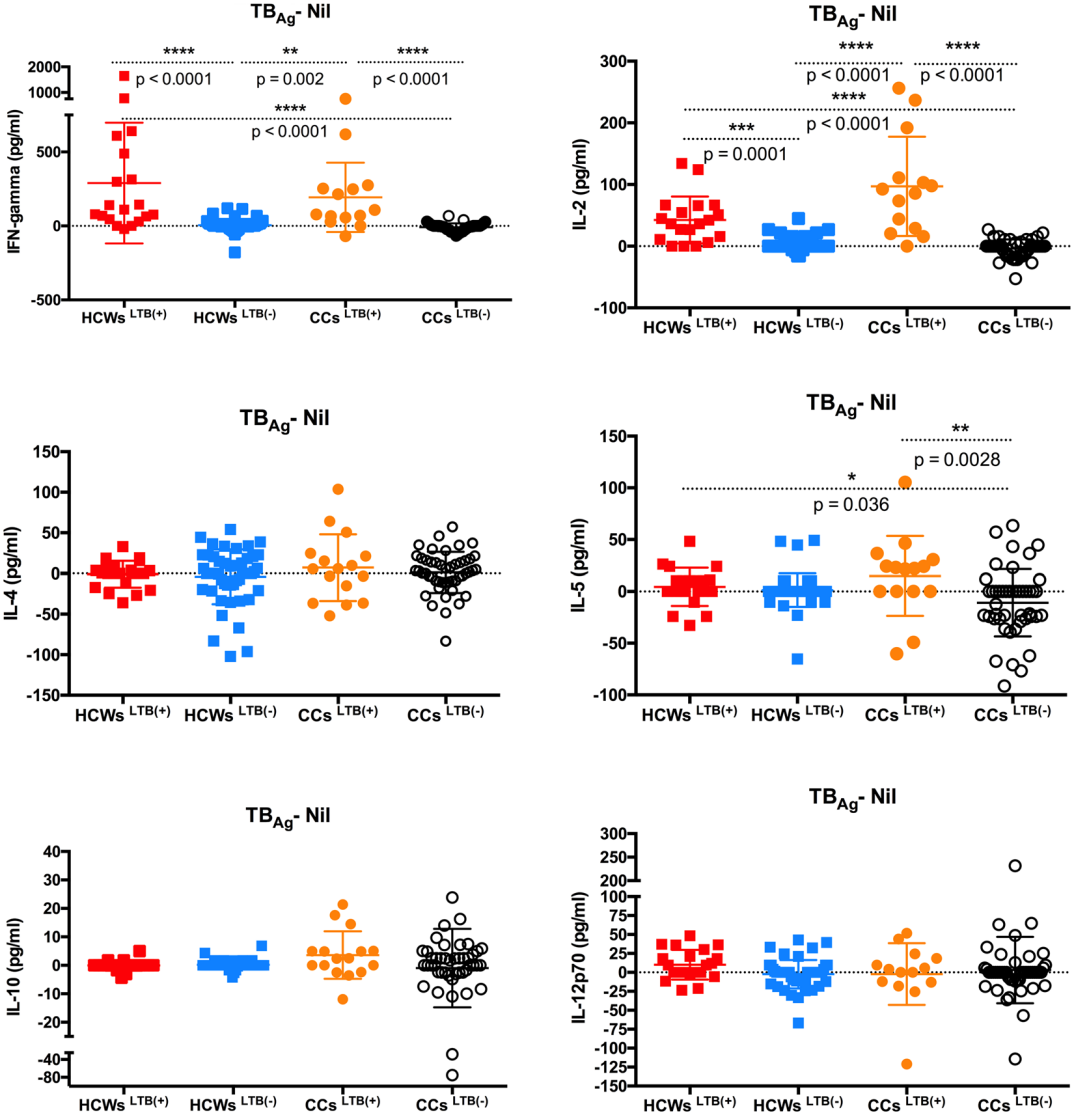
Levels of *M*. *tuberculosis* specific cell mediated cytokines responses (IFN-γ, IL-2, IL-12 (p70), IL-4, IL-5 and IL-10) in the supernatants obtained from the QFT® assays of *M*. *tuberculosis* latently infected (LTB+) and non-infected (LTB−) healthcare workers (HCWs) and community controls (CCs).

The only statistically significant difference in the IL-1β response to *M*. *tuberculosis* antigens was its higher levels in CCs^LTB−^ compared to HCWs^LTB−^ (p = 0.002) (Fig. 3). The levels of IL-8 in response to TB antigens were significantly higher in HCWs^LTB+^ compared to HCWs^LTB−^ (p = 0.003) and CCs^LTB−^ (p = 0.015) (Fig. 3). The IL-8 response was also higher in HCWs^LTB+^ than CCs^LTB+^, but the difference did not reach statistical significance. HCWs^LTB+/−^ showed a weak but positive TNF-α response to *M*. *tuberculosis* antigen stimulation, whereas CCs^LTB+/−^ showed a negative TNF-α response, but only the differences between HCWs^LTB+/−^ and CCs ^LTB−^ were significant (p ≤ 0.015) (Fig. 3). HCWs^LTB+^ and CCs^LTB+^ had significantly higher IFN-γ and IL-2 responses to *M*. *tuberculosis* antigen stimulation than HCWs^LTB−^ and CCs^LTB−^ (p < [0.0001–0.003]) (Fig. 4).

*M*. *tuberculosis* antigen stimulation produced no significant differences between HCW and CC groups for IL-4, IL-10 and IL-12p70. However, the IL-5 concentrations were significantly higher in HCWs^LTB+^ and CCs^LTB+^ than CCs^LTB−^ (p < [0.005–0.04]) (Fig. 4).

#### M. tuberculosis antigen response in latently infected HCWs varied based on active TB exposure gradient

**Figure 5:**
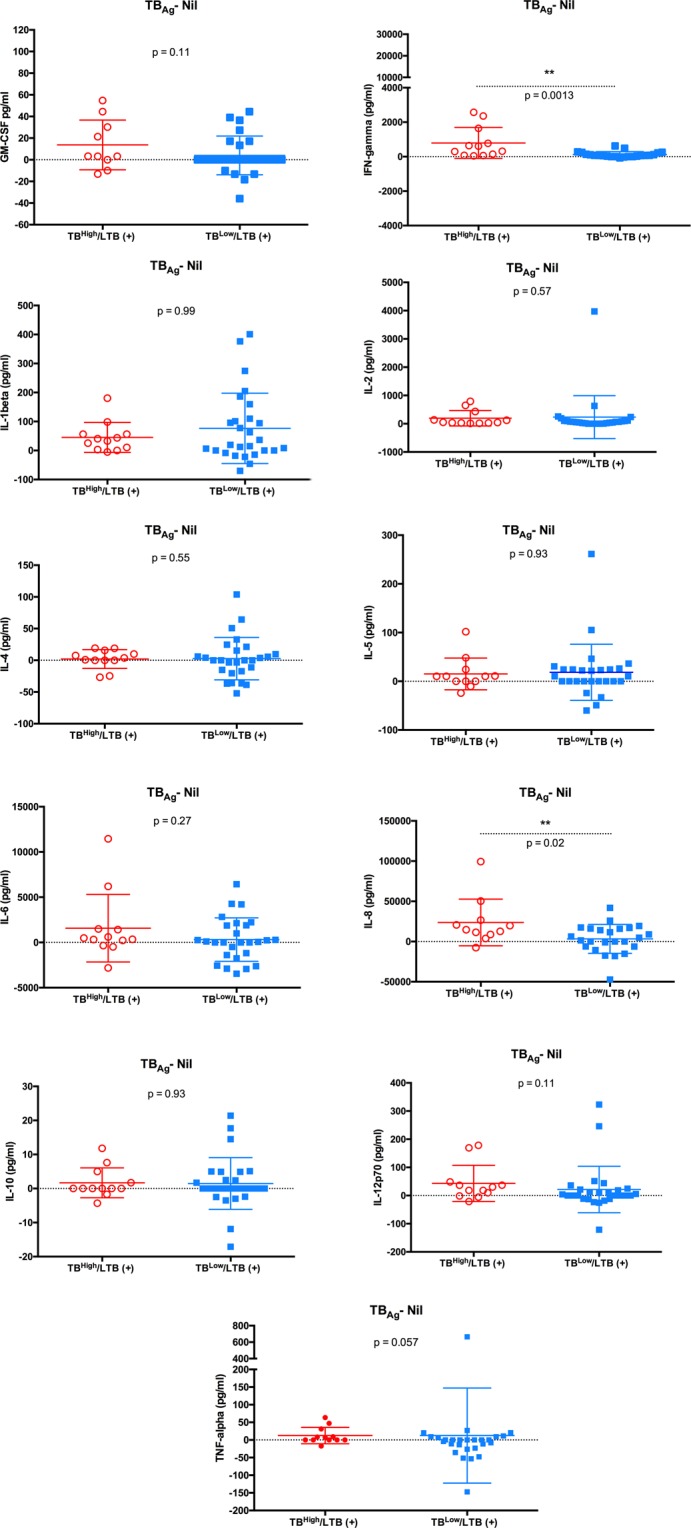
Levels of *M*. *tuberculosis* specific cytokines responses (GM-CSF, IFN-γ, IL-1β, IL-10, IL-12 (p70), IL-2, IL-4, IL-5, IL-6, IL-8, TNF-α) in the supernatants obtained from the QFT® assays of highly (≥3 hours per day) and lowly (not in direct contact with active TB patients) exposed *M*. *tuberculosis* latently infected (LTB+) healthcare workers (HCWs).

To investigate how exposure may affect TB specific cytokines responses, we compared the induction of cytokines in HCWs^LTB+^ with different levels of exposure to active TB. The IFN-γ and IL-8 responses in the highly exposed HCWs^LTB+^, were greater than in the low exposure HCWs^LTB+^ (Mann -Whitney test p = 0.01 and 0.02 respectively) (Fig. 5), but no significant differences were observed for the other cytokines (Fig. 5). Further investigation through non-parametric multiple comparison analysis of (1) highly exposed latently infected HCWs, (2) highly exposed non-infected HCWs, (3) low exposure latently infected HCWs, (4) low exposure non-infected HCWs, (5) latently infected community controls and (6) non-infected community controls showed significant differences in the trends of all cytokine responses to TB specific antigens (p < [0.0001–0.03]) (Supplementary Table).

#### Cytokine responses of HCWs working in contact with active TB patients for 5 to 31 years depended on their M. tuberculosis latent infection status

**Figure 6:**
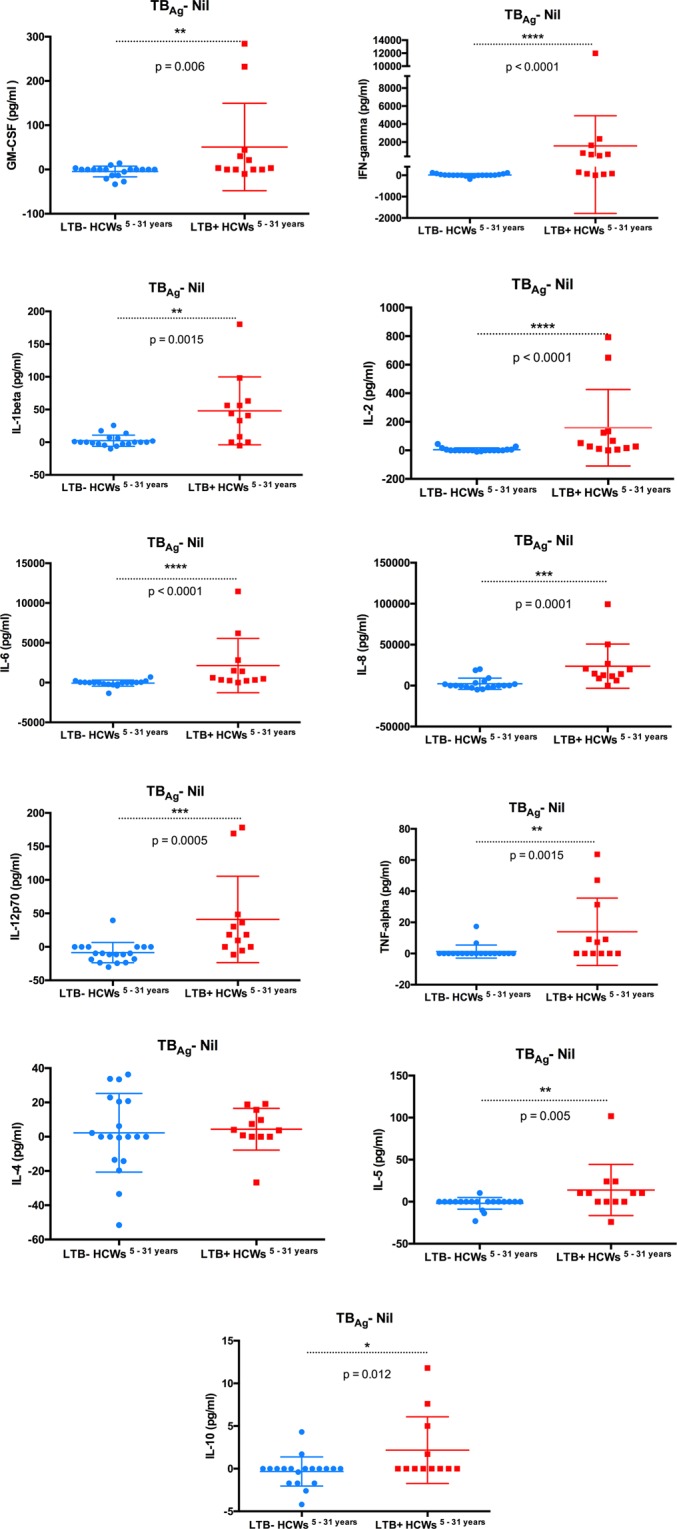
Levels of *M*. *tuberculosis* specific cytokines responses (GM-CSF, IFN-γ, IL-1β, IL-10, IL-12 (p70), IL-2, IL-4, IL-5, IL-6, IL-8, TNF-α) in the supernatants obtained from the QFT® assays of HCWs (*M*. *tuberculosis* latently infected (LTB+) and non-infected (LTB−) working in contact with active TB patients for 5 to 31 years.

In the HCWs who worked in jobs involving high exposure to patients with active TB over periods of 5 to 31 years, the HCWs^LTB+^ showed significantly greater secretion of proinflammatory cytokines GM-CSF, IFN-γ, IL-1β, IL-2, IL-6, IL-8, IL-12p70, and TNF-α in response to *M*. *tuberculosis* antigens than the high exposure HCWs^LTB−^ (p < [0.0001–0.01]) (Fig. 6). In addition, IL-5 and IL-10 (Th2 cytokines) secretion was higher in HCWs^LTB+^ than HCWs^LTB−^. While the differences in the levels of antigen stimulated IL-5 and IL-10 between HCWs^LTB+^ and HCWs^LTB−^ were also significant, the absolute differences in the concentrations were relatively small (Fig. 6). In fact, HCWs^LTB−^ showed virtually no response to antigen stimulation.

## Discussion

Healthcare settings are often high-risk environments for exposure to pathogens and subsequent infections. A recent systematic review focusing on the prevention of *M*. *tuberculosis* infection among HCWs found a 62% prevalence of *M*. *tuberculosis* infection in this population^13^. Our findings illustrate that the risk of latent TB infection was higher in HCWs in direct day-to-day contact with active TB patients (medical doctors, nurses, radiologist and patients’ desk workers) than in HCW with less exposure. We also found that the number of years working in a high exposure healthcare setting also had an influence on the risk of TB infection. Similar observations have been previously reported in Portuguese^14^ and Kenyan^15^ HCWs. In our setting, health workers who spend at least 3 to 4 hours per day in direct contact with active TB patients had greater odds of being infected with *M*. *tuberculosis*. However, while the Kenyan study followed the HCWs to determine their risk of developing active TB, our study could not assess this risk because it did not include prolonged follow-up of HCW participants, which was the principal limitation of our study. We found that men were at higher risk for TB infection, both in the HCWs population and in the community, but the odds of male HCWs being infected by *M*. *tuberculosis* were twice as high as for female HCWs. This could perhaps be explained by their significantly lower BMIs compared to the BMIs of female HCWs^16,17^, but male CCs similarly had greater odds of being infected with *M*. *tuberculosis* than female CCs, despite having comparable BMIs. This appears to confirm that there is a gender inequality in *M*. *tuberculosis* infection, or susceptibility to infection, as has been previously demonstrated in other studies^18,19^. In tuberculosis, differences in social behaviors and biology might also contribute to the gender inequality of infection risks^20,21^.

In our setting, some of the HCWs had been working in high exposure jobs for more than ten years and their immune profiles may be excellent indicators of a protective immune signature. To our knowledge, this is the first study to have investigated TB specific cytokine release in HCWs based on their levels of exposure to active tuberculosis. In unstimulated samples from HCWs, we found a clear trend towards lower levels of both pro- and anti-inflammatory cytokines compared to CCs. Our analysis of cytokine responses to TB specific antigen stimulation revealed that individuals (HCWs and CCs) latently infected with *M*. *tuberculosis* had significantly increased *M*. *tuberculosis* IFN-γ and IL-2 responses compared to non-infected individuals, which is expected, as IFN-γ and IL-2 profiles have been associated with antigenic memory^22–24^. Furthermore, the IL-8 response was significantly higher in HCWs^LTB+^ compared to HCWs^LTB−^ and CCs^LTB−^. Interestingly, HCW^LTB+^ who were highly exposed to active TB patients produced significantly more IFN-γ and IL-8 than HCWs^LTB+^ with low exposure to active TB. Maintaining a high IFN-γ and IL-8 response to *M*. *tuberculosis* infection may be necessary to control TB infection in highly exposed HCWs. Studies have demonstrated that the ability of the macrophage to inhibit the growth of *M*. *tuberculosis* is highly dependent on IFN-γ^3^, and IL-8 is involved in the mycobacterial host-pathogen interaction^25,26^. It has also been shown *in vitro* that *M*. *tuberculosis* antigen induced IL-8 specifically attracted activated human lymphocytes^27^. Moreover, an association between IL-8 gene alleles and human susceptibility to TB disease has been reported^28^. Continuously exposed HCWs^LTB+^ are probably in a state of constant antigen stimulation of the TB specific type-1 cell-mediated immune response.

This idea is also supported by the results of HWCs^LTB+^ in high-exposure jobs for more than five (5) years. These participants have significantly increased *M*. *tuberculosis* antigen stimulated GM-CSF, IFN-γ, IL-1β, IL-2, IL-6, IL-8, IL-12p70, and TNF-α responses compared to HCWs^LTB−^ in high exposure jobs for the same periods of time. The protective cytokines IFN-γ and GM-CSF are thought to be essential for maintaining the ability of mononuclear cells (macrophages, dendritic cells etc.) to control *M*. *tuberculosis* infection^29^. Furthermore, antigen-specific IFN-γ+ IL-2+ CD4+ T cells were found to be effective markers of protection against *M*. *tuberculosis* infection in a murine model^30^.

Considering our observations and the related literature cited here, an effective TB vaccine may need to induce a sustained *M*. *tuberculosis* specific cell-mediated response. This could perhaps be achieved with a vaccine strategy that mimics frequent TB exposure. However, the World Health Organization product characteristic for a new tuberculosis vaccine recommends a minimal number of doses and boosters.

Our data showed that HCWs highly exposed to TB who remained QFT® test negative had low magnitude cytokine responses to stimulation with tuberculosis antigens. This suggests that HCWs highly exposed to TB who remained uninfected may have the ability to clear the infection without developing a detectable adaptive immune response (resistance to TB infection) or to develop highly localized or low magnitude anti-TB immune responses^4^.

In the HCWs who’ve worked in high exposure jobs for more than 5 years, anti-inflammatory and type-2 cell-mediated immune response associated cytokines, including IL-5 and IL-10, were higher in HWCs^LTB+^ compared to HCWs^LTB−^. The IL-10 response could be explained by the need to keep a balance between protective and pathogenic immune responses^29^. IL-5 is a B-cell growth and activation factor^31^ that stimulates the proliferation of lymphocytes and synthesis of antibodies. The borderline positive but significant increase in IL-5 may reflect a controlled B-cell activation and humoral response, which may help control *M*. *tuberculosis* infection^32,33^. Although cell-mediated immunity is key for the control of latent infection, our results support prior evidence suggesting that humoral immunity may also have a role in preventing active TB disease^32,34^.

This study had several limitations, notably, the relatively small number of participants and the lack of long term follow-up. Also, we did not measure the secretion of type-3 cytokines such as IL-17, IL-22 and IL-23^35–38^. Data on the levels of type- 3 cytokines might have provided further evidence of a humoral response contributing to the long-term control of *Mtb* infection^36,39^. It is clear that additional studies, preferably of prospective cohorts with larger samples sizes and variable infection outcomes, are needed to improve our understanding of protective immune responses to TB.

## Conclusion

*M*. *tuberculosis* infection controllers (HWCs^LTB+^) are characterized by an enhanced TB specific cell mediated/proinflammatory cytokine response combined with an induction of selected Th2 type/anti-inflammatory cytokines. In contrast, HCWs with long periods of high TB exposure who appear to be resistant to *M*. *tuberculosis* infection (HWC^LTB−^) did not show an augmented cytokine secretion in response to *M*. *tuberculosis* antigen stimulation. The study of the innate immune response in these individuals may be more appropriate for identifying immunological correlates of resistance to *M*. *tuberculosis* infection.

## Supplementary information

Supplementary Dataset 1
