# Publisher Correction: *M. tuberculosis* infection and antigen specific cytokine response in healthcare workers frequently exposed to tuberculosis

**DOI:** 10.1038/s41598-019-50847-0

**Published:** 2019-10-23

**Authors:** Paulin N. Essone, Marielle Leboueny, Anicet Christel Maloupazoa Siawaya, Amel Kévin Alame-Emane, Oriane Cordelia Aboumegone Biyogo, Patrice Hemery Dapnet Tadatsin, Amandine Mveang Nzoghe, Dimitri Ulrich Essamazokou, Ofilia Mvoundza Ndjindji, Guy-Stéphane Padzys, Selidji Todagbe Agnandji, Howard Takiff, Brigitte Gicquel, Joel Fleury Djoba Siawaya

**Affiliations:** 1 Unité de Recherche et de Diagnostics Spécialisés, Laboratoire National de Santé Publique/Centre Hospitalier Universitaire Mère Enfant Fondation Jeanne EBORI, Libreville, Gabon; 2 Centre de Recherches Médicales de Lambaréné, BP 242 Lambaréné, Gabon; 3 Unité de Génétique Mycobactérienne, Institut Pasteur, Paris, France; 4 Département de Biologie Cellulaire et Physiologie Faculté des Sciences, Université des Sciences et Techniques de Masuku, Franceville, Gabon; 5 Unité de Pathogenomique Mycobactérienne Intégrée, Institut Pasteur, Paris, France; 6 Department of Tuberculosis Control and Prevention, Shenzhen Nanshan Center for Chronic Disease Control, Shenzhen, China; 7 Institut für Tropenmedizin, Universitätsklinikum Tübingen, Tübingen, Germany

Correction to: *Scientific Reports* 10.1038/s41598-019-44294-0, published online 3 June 2019

The original version of this Article contained errors in the affiliation list, where Affiliations 1 and 2 were inverted and the original Affiliation 2 was given as ‘Unité de Recherches et de Diagnostics Spécialisés; Laboratoire National de Santé Publique, Avenue Felix Eboué, BP10 736, Libreville, Gabon’.

The correct Affiliations 1 and 2 are given below:

**Affiliation 1**

Unité de Recherche et de Diagnostics Spécialisés, Laboratoire National de Santé Publique/Centre Hospitalier Universitaire Mère Enfant Fondation Jeanne EBORI, Libreville, Gabon

**Affiliation 2**

Centre de Recherches Médicales de Lambaréné, BP 242, Lambaréné, Gabon

In addition, Patrice Hemery Dapnet Tadatsin was incorrectly affiliated with ‘Hôpital Spécialisé de Nkembo, Libreville, Gabon’. The correct affiliation is listed below:

Unité de Génétique Mycobactérienne, Institut Pasteur, Paris, France

Finally, an additional affiliation for Selidji Todagbe Agnandji was omitted. The correct affiliations for Selidji Todagbe Agnandji are listed below:

Centre de Recherches Médicales de Lambaréné, BP 242, Lambaréné, Gabon

Institut für Tropenmedizin, Universitätsklinikum Tübingen, Tübingen, Germany

These errors have now been corrected in the HTML and PDF versions of this article, and in the accompanying supplementary information file.

