# Supplementary material for: M. tuberculosis infection and antigen specific cytokine response in healthcare workers frequently exposed to tuberculosis
Source: Sci Rep. 2019 Jun 3;9:8201. doi: 10.1038/s41598-019-44294-0 (PMC6547719; doi:10.1038/s41598-019-44294-0)
Supplement: Supplementary file 1 — Supplementary Dataset 1 [file 41598_2019_44294_MOESM1_ESM.pdf]

Supplementary File

M. tuberculosis infection and antigen specific cytokine response in healthcare workers frequently exposed to tuberculosis

Paulin N. Essone[1,2], Marielle Leboueny[1], Anicet Christel Maloupazoa Siawaya[1], Amel Kévin Alame-Emane[1,3], Oriane Cordelia Aboumegone Biyogo[1], Patrice Hemery Dapnet Tadatsin[3], Amandine Mveang Nzoghe[1], Dimitri Ulrich Essamazokou[4], Ofilia Mvoundza Ndjindji[1], Guy-Stéphane Padzys[4], Selidji Todagbe Agnandji[2,7], Howard Takiff [5], Brigitte Gicquel[3,6] & Joel Fleury Djoba Siawaya[1]

1. Unité de Recherche et de Diagnostics Spécialisés, Laboratoire National de Santé Publique / Centre Hospitalier Universitaire Mère Enfant Fondation Jeanne EBORI, Libreville, Gabon

2. Centre de Recherches Médicales de Lambaréné, BP 242, Lambaréné, Gabon

3. Unité de Génétique Mycobactérienne, Institut Pasteur, Paris, France

4. Département de Biologie Cellulaire et Physiologie Faculté des Sciences, Université des Sciences et Techniques de Masuku, Franceville, Gabon

5. Unité de Pathogenomique Mycobactérienne Intégrée, Institut Pasteur, Paris, France

6. Department of Tuberculosis Control and Prevention, Shenzhen Nanshan Center for Chronic Disease Control, Shenzhen, China

7. Institut für Tropenmedizin, Universitätsklinikum Tübingen, Tübingen, Germany

Supplementary File

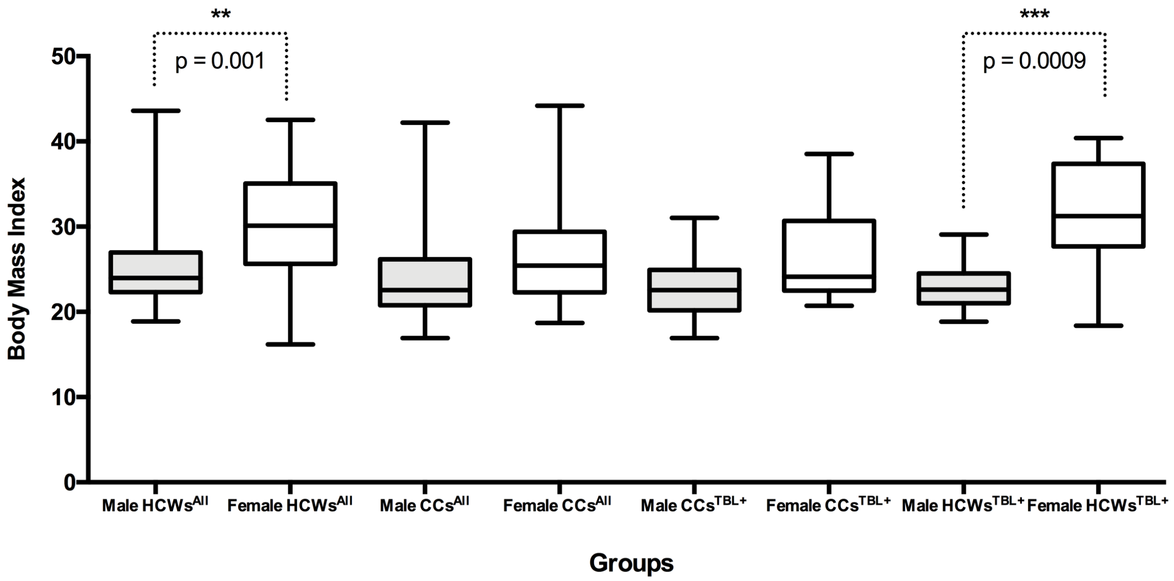

Supplementary figure. Body mass index (BMI) of females and males healthcare workers (HCWs) and community controls (CCs)

Supplementary File

Supplementary table: Multi comparison analysis of TB specific antigens cytokines responses in of (1) highly exposed latently infected HCWs, (2) highly exposed non-infected HCWs, (3) low exposure latently infected HCWs, (4) low exposure non-infected HCWs, (5) latently infected community controls and (6) non-infected community controls.

GM-CSF					IFN-gamma			
**Kruskal-Wallis test**					**Kruskal-Wallis test**			
P value	< 0,0001				P value	< 0,0001		
**Dunn's multiple comparisons test**								
	Mean rank diff,	Significant?	Summary	Adjusted P Value	Mean rank diff,	Significant?	Summary	Adjusted P Value
TB^{High}/LTB (+) vs. TB^{High}/LTB (-)	30,61	No	ns	0,257	61,02	**Yes**	****	0,257
TB^{High}/LTB (+) vs. TB^{Low}/LTB (+)	11,8	No	ns	> 0,9999	9,54	No	ns	> 0,9999
TB^{High}/LTB (+) vs. TB^{Low}/LTB (-)	38,64	**Yes**	*	**0,0342**	75,17	**Yes**	****	**0,0342**
TB^{High}/LTB (+) vs. CC/LTB (+)	-6,872	No	ns	> 0,9999	14,72	No	ns	> 0,9999
TB^{High}/LTB (+) vs. CC/LTB (-)	7,83	No	ns	> 0,9999	56,68	**Yes**	****	> 0,9999
TB^{High}/LTB (-) vs. TB^{Low}/LTB (+)	-18,82	No	ns	> 0,9999	-51,48	**Yes**	**	> 0,9999
TB^{High}/LTB (-) vs. TB^{Low}/LTB (-)	8,025	No	ns	> 0,9999	14,15	No	ns	> 0,9999
TB^{High}/LTB (-) vs. CC/LTB (+)	-37,49	**Yes**	*	**0,0155**	-46,3	**Yes**	**	**0,0155**
TB^{High}/LTB (-) vs. CC/LTB (-)	-22,78	No	ns	0,1816	-4,341	No	ns	0,1816
TB^{Low}/LTB (+) vs. TB^{Low}/LTB (-)	26,84	No	ns	0,7248	65,63	**Yes**	****	0,7248
TB^{Low}/LTB (+) vs. CC/LTB (+)	-18,67	No	ns	> 0,9999	5,184	No	ns	> 0,9999
TB^{Low}/LTB (+) vs. CC/LTB (-)	-3,968	No	ns	> 0,9999	47,14	**Yes**	**	> 0,9999
TB^{Low}/LTB (-) vs. CC/LTB (+)	-45,51	**Yes**	***	**0,0008**	-60,45	**Yes**	****	**0,0008**
TB^{Low}/LTB (-) vs. CC/LTB (-)	-30,81	**Yes**	**	**0,0072**	-18,49	No	ns	**0,0072**
CC/LTB (+) vs. CC/LTB (-)	14,7	No	ns	> 0,9999	41,96	**Yes**	**	> 0,9999

Supplementary File

	IL-1beta				IL-2			
	**Kruskal-Wallis test**<br>P value < 0,0001				**Kruskal-Wallis test**<br>P value < 0,0001			
	Mean rank diff,	Significant?	Summary	Adjusted P Value	Mean rank diff,	Significant?	Summary	Adjusted P Value
TB^{High}/LTB (+) vs. TB^{High}/LTB (-)	30,95	No	ns	0,3236	58,3	**Yes**	***	**0,0002**
TB^{High}/LTB (+) vs. TB^{Low}/LTB (+)	0,4798	No	ns	> 0,9999	5,657	No	ns	> 0,9999
TB^{High}/LTB (+) vs. TB^{Low}/LTB (-)	27,82	No	ns	0,5211	61,71	**Yes**	****	< 0,0001
TB^{High}/LTB (+) vs. CC/LTB (+)	-35	No	ns	0,2029	-5,642	No	ns	> 0,9999
TB^{High}/LTB (+) vs. CC/LTB (-)	-32,68	No	ns	0,1107	47,03	**Yes**	**	**0,0016**
TB^{High}/LTB (-) vs. TB^{Low}/LTB (+)	-30,47	No	ns	0,5186	-52,64	**Yes**	**	**0,0032**
TB^{High}/LTB (-) vs. TB^{Low}/LTB (-)	-3,128	No	ns	> 0,9999	3,417	No	ns	> 0,9999
TB^{High}/LTB (-) vs. CC/LTB (+)	-65,95	**Yes**	****	**< 0,0001**	-63,94	**Yes**	****	**< 0,0001**
TB^{High}/LTB (-) vs. CC/LTB (-)	-63,63	**Yes**	****	**< 0,0001**	-11,26	No	ns	> 0,9999
TB^{Low}/LTB (+) vs. TB^{Low}/LTB (-)	27,34	No	ns	0,7987	56,06	**Yes**	***	**0,001**
TB^{Low}/LTB (+) vs. CC/LTB (+)	-35,48	No	ns	0,2792	-11,3	No	ns	> 0,9999
TB^{Low}/LTB (+) vs. CC/LTB (-)	-33,16	No	ns	0,1837	41,38	**Yes**	*	**0,0249**
TB^{Low}/LTB (-) vs. CC/LTB (+)	-62,82	**Yes**	****	**< 0,0001**	-67,35	**Yes**	****	**< 0,0001**
TB^{Low}/LTB (-) vs. CC/LTB (-)	-60,5	**Yes**	****	**< 0,0001**	-14,68	No	ns	> 0,9999
CC/LTB (+) vs. CC/LTB (-)	2,321	No	ns	> 0,9999	52,68	**Yes**	****	**< 0,0001**

Supplementary File

	IL-4				IL-5			
	**Kruskal-Wallis test**				**Kruskal-Wallis test**			
	P value	< 0,0001			P value	< 0,0001		
	Mean rank diff,	Significant?	Summary	Adjusted P Value	Mean rank diff,	Significant?	Summary	Adjusted P Value
TB^{High}/LTB (+) vs. TB^{High}/LTB (-)	-14,66	No	ns	> 0,9999	16,23	No	ns	> 0,9999
TB^{High}/LTB (+) vs. TB^{Low}/LTB (+)	-23,11	No	ns	> 0,9999	5,54	No	ns	> 0,9999
TB^{High}/LTB (+) vs. TB^{Low}/LTB (-)	-18,34	No	ns	> 0,9999	26,11	No	ns	0,6877
TB^{High}/LTB (+) vs. CC/LTB (+)	-48,15	**Yes**	*	**0,0114**	-30,84	No	ns	0,4252
TB^{High}/LTB (+) vs. CC/LTB (-)	-49,99	**Yes**	***	**0,0007**	-15,66	No	ns	> 0,9999
TB^{High}/LTB (-) vs. TB^{Low}/LTB (+)	-8,447	No	ns	> 0,9999	-10,69	No	ns	> 0,9999
TB^{High}/LTB (-) vs. TB^{Low}/LTB (-)	-3,676	No	ns	> 0,9999	9,883	No	ns	> 0,9999
TB^{High}/LTB (-) vs. CC/LTB (+)	-33,49	No	ns	0,0787	-47,07	**Yes**	***	**0,001**
TB^{High}/LTB (-) vs. CC/LTB (-)	-35,33	**Yes**	**	**0,0032**	-31,89	**Yes**	*	**0,0101**
TB^{Low}/LTB (+) vs. TB^{Low}/LTB (-)	4,771	No	ns	> 0,9999	20,57	No	ns	> 0,9999
TB^{Low}/LTB (+) vs. CC/LTB (+)	-25,04	No	ns	> 0,9999	-36,38	No	ns	0,2257
TB^{Low}/LTB (+) vs. CC/LTB (-)	-26,88	No	ns	0,6626	-21,2	No	ns	> 0,9999
TB^{Low}/LTB (-) vs. CC/LTB (+)	-29,81	No	ns	0,1712	-56,95	**Yes**	****	**< 0,0001**
TB^{Low}/LTB (-) vs. CC/LTB (-)	-31,65	**Yes**	**	**0,0095**	-41,77	**Yes**	****	**< 0,0001**
CC/LTB (+) vs. CC/LTB (-)	-1,841	No	ns	> 0,9999	15,18	No	ns	> 0,9999

Supplementary File

	IL-6				IL-8			
	**Kruskal-Wallis test**				**Kruskal-Wallis test**			
	P value	< 0,0001			P value	< 0,0001		
	Mean rank diff,	Significant?	Summary	Adjusted P Value	Mean rank diff,	Significant?	Summary	Adjusted P Value
TB^{High}/LTB (+) vs. TB^{High}/LTB (-)	15,48	No	ns	> 0,9999	38,29	No	ns	0,0677
TB^{High}/LTB (+) vs. TB^{Low}/LTB (+)	-10,26	No	ns	> 0,9999	-10,74	No	ns	> 0,9999
TB^{High}/LTB (+) vs. TB^{Low}/LTB (-)	24,64	No	ns	0,958	35,15	No	ns	0,1155
TB^{High}/LTB (+) vs. CC/LTB (+)	-25,78	No	ns	> 0,9999	-1,057	No	ns	> 0,9999
TB^{High}/LTB (+) vs. CC/LTB (-)	-19,99	No	ns	> 0,9999	-1,295	No	ns	> 0,9999
TB^{High}/LTB (-) vs. TB^{Low}/LTB (+)	-25,73	No	ns	> 0,9999	-49,03	Yes	*	0,0102
TB^{High}/LTB (-) vs. TB^{Low}/LTB (-)	9,161	No	ns	> 0,9999	-3,143	No	ns	> 0,9999
TB^{High}/LTB (-) vs. CC/LTB (+)	-41,26	**Yes**	**	**0,0088**	-39,35	**Yes**	*	**0,0159**
TB^{High}/LTB (-) vs. CC/LTB (-)	-35,47	**Yes**	**	**0,003**	-39,59	**Yes**	***	**0,0006**
TB^{Low}/LTB (+) vs. TB^{Low}/LTB (-)	34,9	No	ns	0,2173	45,89	**Yes**	*	**0,0179**
TB^{Low}/LTB (+) vs. CC/LTB (+)	-15,52	No	ns	> 0,9999	9,681	No	ns	> 0,9999
TB^{Low}/LTB (+) vs. CC/LTB (-)	-9,731	No	ns	> 0,9999	9,442	No	ns	> 0,9999
TB^{Low}/LTB (-) vs. CC/LTB (+)	-50,42	**Yes**	***	**0,0003**	-36,21	**Yes**	*	**0,0293**
TB^{Low}/LTB (-) vs. CC/LTB (-)	-44,63	**Yes**	****	**< 0,0001**	-36,45	**Yes**	**	**0,0011**
CC/LTB (+) vs. CC/LTB (-)	5,79	No	ns	> 0,9999	-0,2386	No	ns	> 0,9999

Supplementary File

	IL-10				IL-12p70			
	**Kruskal-Wallis test**				**Kruskal-Wallis test**			
	P value	< 0,0001			P value	< 0,0393		
	Mean rank diff,	Significant?	Summary	Adjusted P Value	Mean rank diff,	Significant?	Summary	Adjusted P Value
TB^{High}/LTB (+) vs. TB^{High}/LTB (-)	6,223	No	ns	> 0,9999	24,86	No	ns	0,9534
TB^{High}/LTB (+) vs. TB^{Low}/LTB (+)	-9,708	No	ns	> 0,9999	-4,843	No	ns	> 0,9999
TB^{High}/LTB (+) vs. TB^{Low}/LTB (-)	5,938	No	ns	> 0,9999	15,84	No	ns	> 0,9999
TB^{High}/LTB (+) vs. CC/LTB (+)	-57,9	**Yes**	***	**0,0002**	-9,923	No	ns	> 0,9999
TB^{High}/LTB (+) vs. CC/LTB (-)	-47,84	**Yes**	***	**0,0004**	5,136	No	ns	> 0,9999
TB^{High}/LTB (-) vs. TB^{Low}/LTB (+)	-15,93	No	ns	> 0,9999	-29,71	No	ns	0,5786
TB^{High}/LTB (-) vs. TB^{Low}/LTB (-)	-0,286	No	ns	> 0,9999	-9,027	No	ns	> 0,9999
TB^{High}/LTB (-) vs. CC/LTB (+)	-64,12	**Yes**	****	**< 0,0001**	-34,79	No	ns	0,053
TB^{High}/LTB (-) vs. CC/LTB (-)	-54,07	**Yes**	****	**< 0,0001**	-19,73	No	ns	0,5605
TB^{Low}/LTB (+) vs. TB^{Low}/LTB (-)	15,65	No	ns	> 0,9999	20,68	No	ns	> 0,9999
TB^{Low}/LTB (+) vs. CC/LTB (+)	-48,19	**Yes**	*	**0,0235**	-5,08	No	ns	> 0,9999
TB^{Low}/LTB (+) vs. CC/LTB (-)	-38,14	No	ns	0,0723	9,98	No	ns	> 0,9999
TB^{Low}/LTB (-) vs. CC/LTB (+)	-63,83	**Yes**	****	**< 0,0001**	-25,76	No	ns	0,4179
TB^{Low}/LTB (-) vs. CC/LTB (-)	-53,78	**Yes**	****	**< 0,0001**	-10,7	No	ns	> 0,9999
CC/LTB (+) vs. CC/LTB (-)	10,05	No	ns	> 0,9999	15,06	No	ns	> 0,9999

Supplementary File

	TNF-alpha			
	Kruskal-Wallis test			
	P value	< 0,0001		
	Mean rank diff,	Significant?	Summary	Adjusted P Value
TB^{High}/LTB (+) vs. TB^{High}/LTB (-)	19,48	No	ns	> 0,9999
TB^{High}/LTB (+) vs. TB^{Low}/LTB (+)	-6,556	No	ns	> 0,9999
TB^{High}/LTB (+) vs. TB^{Low}/LTB (-)	28,1	No	ns	0,3911
TB^{High}/LTB (+) vs. CC/LTB (+)	-19,03	No	ns	> 0,9999
TB^{High}/LTB (+) vs. CC/LTB (-)	-19,67	No	ns	> 0,9999
TB^{High}/LTB (-) vs. TB^{Low}/LTB (+)	-26,03	No	ns	0,8681
TB^{High}/LTB (-) vs. TB^{Low}/LTB (-)	8,627	No	ns	> 0,9999
TB^{High}/LTB (-) vs. CC/LTB (+)	-38,51	**Yes**	*	**0,0109**
TB^{High}/LTB (-) vs. CC/LTB (-)	-39,15	**Yes**	***	**0,0002**
TB^{Low}/LTB (+) vs. TB^{Low}/LTB (-)	34,66	No	ns	0,1587
TB^{Low}/LTB (+) vs. CC/LTB (+)	-12,48	No	ns	> 0,9999
TB^{Low}/LTB (+) vs. CC/LTB (-)	-13,11	No	ns	> 0,9999
TB^{Low}/LTB (-) vs. CC/LTB (+)	-47,14	**Yes**	***	**0,0004**
TB^{Low}/LTB (-) vs. CC/LTB (-)	-47,77	**Yes**	****	**< 0,0001**
CC/LTB (+) vs. CC/LTB (-)	-0,6392	No	ns	> 0,9999
